# Cross-Identity Interaction Transformer for Facial Age Estimation

**DOI:** 10.3390/s26103157

**Published:** 2026-05-16

**Authors:** Yiming Ma, Chunlong Hu, Changbin Shao, Hualong Yu

**Affiliations:** School of Computer Science, Jiangsu University of Science and Technology, Zhenjiang 212003, China; 232241807524@stu.just.edu.cn (Y.M.); shaocb@just.edu.cn (C.S.); yuhualong@just.edu.cn (H.Y.)

**Keywords:** facial age estimation, cross-identity interaction, prior-guided axial cross-image attention

## Abstract

Despite the remarkable progress being made in the study of human facial age estimation, it is still a challenging problem. The main problem lies in the large intra-age appearance variations among different individuals. Sometimes, these variations can even exceed the inter-age appearance variations of the same individual. To address this problem, we construct a cross-identity image sequence for each query image and reformulate age estimation as a multi-image learning task. This provides a basis for learning common age-related cues across identities. Based on this formulation, we propose the Cross-Identity Interaction Transformer (CIIT) for age estimation. The CIIT first extracts multi-scale aging cues through a cross-scale embedding (CSE) module to preserve age evidence from fine textures to coarse structural changes. Secondly, to progressively enhance facial features and capture shared facial characteristics from cross-identity references, intra-image feature attention (IFA) and prior-guided axial cross-image attention (PG-ACIA) operate alternately within each Transformer block. IFA refines local age-discriminative representations within each image, while PG-ACIA uses multi-scale edge priors to guide cross-image interaction toward age-sensitive regions such as wrinkles. Finally, an anchored regression network (ARN) predicts age through a soft-weighted combination of multiple linear regressors for robust age estimation under diverse facial aging patterns. Experiments on four benchmark datasets, namely MORPH Album II, MegaAge-Asian, FG-NET and Adience, demonstrate that the proposed method achieves superior performance across multiple evaluation metrics, validating the effectiveness of the CIIT in capturing shared facial characteristics.

## 1. Introduction

Facial age estimation aims to predict chronological age from facial images and has broad applications in intelligent surveillance, human–computer interaction and cross-age face recognition. With the rapid advancement of deep learning, CNN-based and Transformer-based methods have achieved substantial progress in facial age estimation through continuous improvements in feature representation and classification. Nevertheless, these methods estimate age from a single facial image and therefore fail to capture the shared facial characteristics among different individuals with the same age.

**Figure 1:**
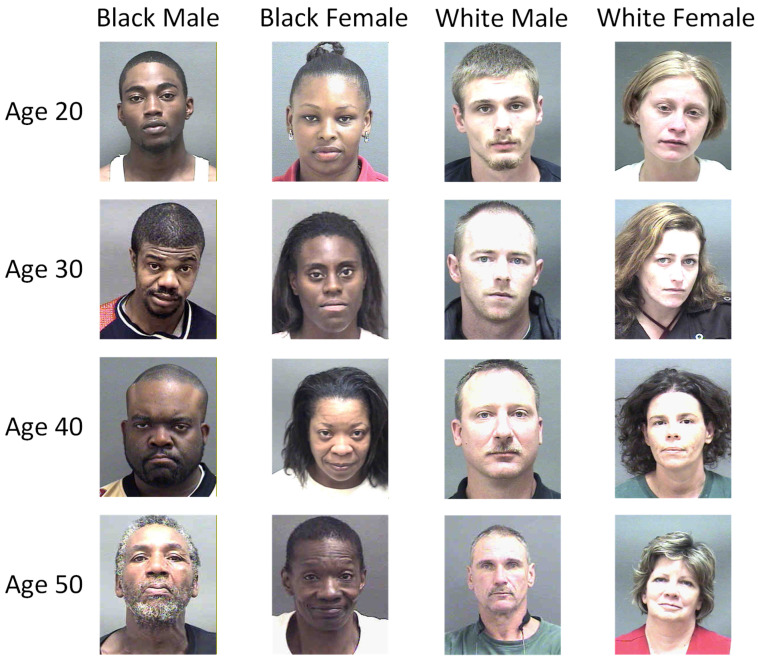
Facial images of different individuals at the same chronological age.

**Figure 2:**
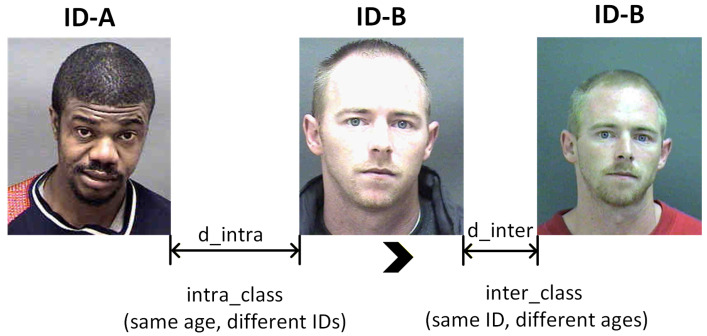
Illustration of intra-class and inter-class distances in the feature space of facial age estimation.

As illustrated in Figure 1, across the age range from 20 to 50 years, individuals of the same chronological age can exhibit markedly different degrees of facial aging owing to differences in sex, ethnicity, genetics, lifestyle and other factors. Some faces remain relatively smooth, whereas others show clearly visible crow’s feet and nasolabial folds. Such variations reflect the high heterogeneity of appearance among same-age faces, which is the fundamental challenge of facial age estimation. As shown in Figure 2, in the feature embedding spaces learned by mainstream architectures such as ResNet, the intra-age distance $d_{\mathrm{intra}}$ of two images from different individuals sometimes exceeds the inter-age distance $d_{\mathrm{inter}}$ of two images from the same individual. As a result, age estimation is easily interfered with by information that is unrelated to age, such as identity, which blurs age-discriminative boundaries and limits prediction accuracy.

Further analysis reveals two key aspects of facial age estimation. Firstly, aging cues appear at multiple spatial scales, from fine-grained wrinkles to coarse structural changes. This highlights the importance of multi-scale feature modeling. Secondly, single-image-based methods estimate age from only one facial image and therefore cannot capture the shared facial characteristics among different individuals of the same age. Using same-age reference images and explicit cross-identity interaction helps to capture such shared characteristics and reduce identity interference.

Motivated by the above analysis, we propose the Cross-Identity Interaction Transformer (CIIT), which combines multi-scale feature extraction with cross-identity interaction to learn shared age-related characteristics. Specifically, cross-scale embedding (CSE) captures aging cues at multiple spatial scales through multi-receptive-field parallel convolutions. Each Transformer block alternates two complementary operations. Intra-image feature attention (IFA) strengthens the local age-discriminative cues within each image, whereas prior-guided axial cross-image attention (PG-ACIA) performs row–column interaction across the image sequence to align corresponding age-sensitive features across images. Its row and column branches are designed to exploit the directional continuity of age-sensitive facial structures, which helps to align shared patterns across different identities. Finally, an anchored regression network (ARN) produces the age estimate through a soft-weighted combination of multiple linear regressors, making the final prediction less sensitive to diverse aging patterns and less reliable reference cues.

The main contributions of this paper are as follows:

(1) We propose a cross-identity image-sequence framework that reformulates age estimation from single-image-based methods into a multi-image learning task. By constructing a same-age reference sequence for each query image, the framework provides the basis for learning shared facial characteristics across identities and mitigating identity interference.
(2) We propose PG-ACIA for capturing shared facial characteristics. It uses row–column axial attention because many age-sensitive facial structures, such as forehead lines and nasolabial folds, extend mainly along horizontal or vertical directions, making shared patterns easier to align across identities.
(3) Extensive experiments on MORPH Album II, MegaAge-Asian, FG-NET and Adience demonstrate the effectiveness of the CIIT. The proposed method achieves competitive performance across multiple evaluation metrics, validating the effectiveness of learning shared facial characteristics through cross-identity references.

## 2. Related Work

### 2.1. Deep Learning Methods

Early facial age estimation mainly relied on handcrafted descriptors such as LBP [1] and Gabor filters [2] with statistical prediction models. Although effective under controlled conditions, these methods generalize poorly to unconstrained settings. With deep learning, feature extraction methods for facial age estimation have mainly developed along two lines, CNN-based methods and Transformer-based methods.

CNN-based methods improve age estimation through large-scale pretraining, ordinal supervision, lightweight design and feature enhancement. DEX [3] demonstrates the value of large-scale pretraining for age prediction. Ranking-CNN [4] introduces multiple binary classifiers to encode ordinal relationships among age labels. SSR-Net [5] and C3AE [6] further improve lightweight age estimation through soft stagewise regression and compact cascaded design, respectively. FPN [7], SE [8], CBAM [9] and FP-Age [10] further enhance facial representations through cross-level fusion, channel reweighting, spatial attention and face-parsing-guided region selection, respectively.

Transformer-based methods further strengthen contextual modeling. ViT [11] treats an image as a patch sequence and models long-range dependencies through self-attention. Swin Transformer [12] introduces shifted windows for hierarchical feature modeling. DAA [13] incorporates delta-age encoding. SwinFace [14] introduces multi-task attribute prediction. Recent RGB-T vision architectures also adopt divide-and-conquer triple-flow designs to separately model modality-specific and complementary cues for robust fusion [15]. CSCS-Swin [16] models cross-window interaction. However, these methods still rely on single-image prediction and cannot explicitly capture the shared facial characteristics among different individuals of the same age.

### 2.2. Age Label Encoding Methods

Age label encoding methods improve facial age estimation by redesigning the target representation used in training. The existing methods mainly fall into two categories, including label distribution learning and ordinal regression.

Label distribution learning represents age as a distribution over neighboring labels rather than a single target. LDL [17] introduced Gaussian-centered label distributions to model semantic correlations between neighboring ages. DLDL-v2 [18] further combines distribution learning with expectation regression. MV-Loss [19] jointly models the mean and variance of the target distribution. AVDL [20] adapts the distribution width to each sample. OLDL [21] further incorporates ordinal structure into label distribution learning.

Ordinal regression methods model age as an ordered prediction problem. CORAL [22] enforces rank-consistent binary outputs through weight sharing. MWR [23] reformulates age prediction as a sliding local subproblem to smooth transitions between adjacent ages. BridgeNet [24] decomposes global regression into multiple local regressors to handle heterogeneous aging patterns across age ranges.

These methods improve age estimation mainly by redesigning supervision signals. However, they still predict age from each face independently and do not explicitly model the shared facial characteristics among different individuals of the same age.

### 2.3. Comparative Learning Methods

Comparative learning methods seek to go beyond single-image-based methods by exploiting relations across multiple samples. The existing approaches mainly fall into two categories, constraint-level methods and input-level methods.

Constraint-level methods introduce cross-sample relations through loss functions or graph structures. OrdCon [25] aligns feature trajectories with the natural aging direction through contrastive soft proxy matching. GroupFace [26] addresses intra-class imbalance with a group-aware attention graph and a margin loss. LRA-GNN [27] models latent inter-landmark relations through random-walk-augmented graph convolution. However, these methods do not establish explicit spatial correspondences across different individuals.

Input-level methods incorporate multiple images directly into the model input. MiVOLO [28] demonstrates the benefit of multi-stream input by jointly processing face and body images of the same subject, but its streams provide complementary cues within one subject rather than across different identities. GFL [29] performs intra-group lateral comparison through a visual permutation-recovery task over shuffled same-age sequences, but the comparison is limited to sequence restoration and does not establish feature-level spatial alignment.

These studies show the value of relational modeling across multiple samples, but spatial alignment of corresponding features across different identities of the same age is still lacking.

## 3. Methodology

### 3.1. Overall Architecture

**Figure 3:**
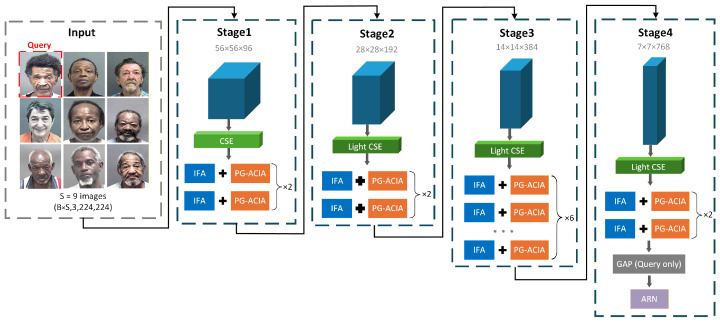
Overall architecture of the proposed CIIT network.

As illustrated in Figure 3, the CIIT reformulates age estimation as a multi-image interaction task based on a cross-identity image sequence. For each query image, same-age reference images of diverse identities are assembled into an input sequence. This enables the model to learn shared facial characteristics through cross-identity interaction rather than overfitting to the appearance of any single identity. The framework is constructed from three complementary dimensions, namely multi-scale feature extraction, an alternating dual-attention mechanism and anchored regression.

The CIIT adopts a four-stage pyramid backbone with Transformer block depths of [2,2,6,2]. Across the four stages, the channel dimension progressively expands from 96 to 768, while the spatial resolution decreases from 56×56 to 7×7. At each stage, CSE uses multi-receptive-field parallel convolutions to extract aging cues at different spatial granularities while jointly encoding features and downsampling the feature map. The resulting features are then fed into a sequence of Transformer blocks, where IFA and PG-ACIA operate alternately. IFA refines local feature representations within each image, while PG-ACIA uses multi-scale edge priors to guide sparse axial interactions across the image sequence. Together, the two mechanisms form an iterative interaction process. After the four-stage backbone, global average pooling is applied only to the query-image features. The resulting feature vector is then fed into ARN for final age prediction.

### 3.2. Cross-Scale Embedding Module

**Figure 4:**
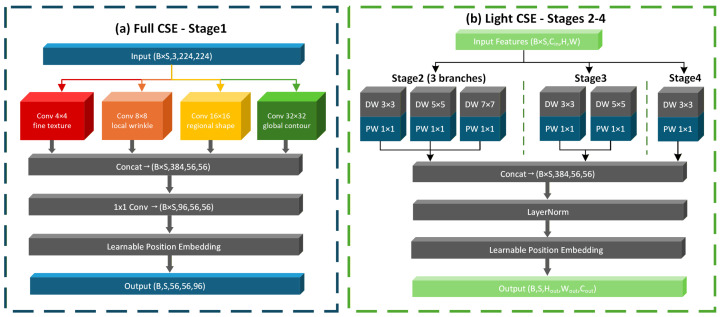
Architecture of the CSE module. (**a**) Full CSE for Stage 1. (**b**) Light CSE for Stages 2–4.

Facial aging cues appear at different spatial scales. Fine details such as wrinkles and skin pores require small receptive fields, whereas coarser changes such as under-eye sagging, cheek drooping and overall facial structure changes require larger ones. Although multi-scale feature extraction is widely used, most existing methods apply it only at the initial stage and keep the same kernel design in deeper layers. CSE addresses this limitation by using stage-adaptive multi-receptive-field parallel convolutions across the backbone, as shown in Figure 4.

In Stage 1, the input is the raw RGB image, where the need for multi-scale modeling is greatest. As shown in Figure 4a, full CSE uses four parallel standard convolution branches with kernel sizes of 4×4, 8×8, 16×16, and 32×32, a unified stride of 4, and 96 output channels per branch. The 4×4 branch captures ultra-fine textures such as skin micro-wrinkles and pores. The 8×8 branch captures local wrinkle patterns such as crow’s feet and nasolabial folds. The 16×16 branch models structural changes in facial subregions such as the orbital area and the nasolabial groove. The 32×32 branch captures larger changes in overall facial structure. The outputs of the four branches are concatenated along the channel dimension, passed through a 1×1 convolution to match the target channel count and then augmented with learnable position embeddings.

(1) $$F_{\mathrm{fused}}={\mathrm{Conv}}_{1\times 1}\;\left(\mathrm{Concat}(F_{4},\;F_{8},\;F_{16},\;F_{32})\right)$$

In Stages 2 through 4, the spatial resolution decreases while the channel dimension increases. Using full CSE unchanged at these stages would sharply increase the parameter count. Light CSE therefore replaces standard convolutions with depthwise separable convolutions. Each branch uses a depthwise convolution followed by a 1×1 pointwise convolution, reducing parameters while preserving cross-scale modeling capacity, as shown in Figure 4b. The kernel sizes are also reduced as the spatial resolution decreases. At Stage 2, where the feature map resolution is 28×28, three parallel branches with kernels [3×3,5×5,7×7] are used to capture mid-level aging cues. At Stage 3, where the resolution is 14×14, two branches with kernels [3×3,5×5] are used. At Stage 4, where the features are already highly abstract, a single 3×3 depthwise separable branch is sufficient. This progressive lightweight design reduces the parameter count of the embedding modules in Stages 2–4 to 1.8 M, about 85% lower than using full CSE at these stages.

### 3.3. Dual-Attention Mechanism

**Figure 5:**
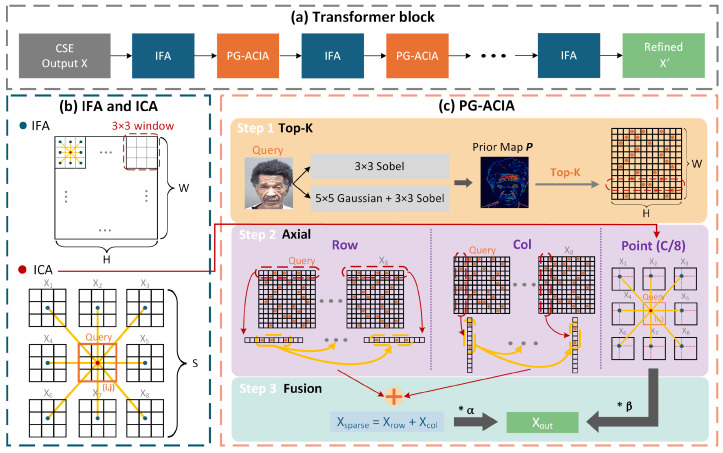
Illustration of the dual-attention mechanism. (**a**) Transformer block. (**b**) IFA and ICA. (**c**) PG-ACIA.

Cross-image interaction in facial age estimation involves two key issues. Local features should first be refined within each image because insufficiently refined representations lead to inaccurate cross-image alignment. Dense cross-image attention is also computationally expensive, especially on high-resolution feature maps. Accordingly, each Transformer block alternates IFA and PG-ACIA, as illustrated in Figure 5a. IFA performs local intra-image refinement, whereas PG-ACIA performs efficient cross-image interaction.

#### 3.3.1. Intra-Image Feature Attention

Aging cues such as crow’s feet and local skin texture gradients are highly localized. Global self-attention introduces many irrelevant long-range dependencies and can blur local age-discriminative patterns. As shown in the upper part of Figure 5b, IFA applies non-overlapping 3×3 window-based self-attention independently to each image. This design refines local age-discriminative features within compact neighborhoods without mixing information across the image sequence. It therefore provides cleaner intra-image representations for subsequent cross-image interaction.

#### 3.3.2. Inter-Image Comparison Attention

The most direct form of cross-image interaction is inter-image comparison attention (ICA). For an input sequence $X\in R^{B\times S\times H\times W\times C}$, ICA applies attention across the *S* image features at the same spatial position (i,j), as shown in the lower part of Figure 5b. This operation directly aligns corresponding spatial features from different images and establishes explicit cross-image correspondences at every location. However, this formulation has complexity $O(HW\cdot S^{2}\cdot C)$ and is therefore costly on high-resolution feature maps. PG-ACIA is introduced to reduce this cost through prior-guided sparse selection and axial decomposition. To preserve cross-image interaction outside the selected sparse set, PG-ACIA retains a lightweight point branch. It follows the same interaction pattern as ICA at each spatial position but uses a reduced hidden dimension of C/8.

#### 3.3.3. Prior-Guided Axial Cross-Image Attention

PG-ACIA introduces two improvements over ICA, as illustrated in Figure 5c.

The first is multi-scale prior-guided sparse position selection. Age-sensitive cues tend to cluster around strong local gradients such as wrinkles and under-eye contours, whereas large smooth regions contribute less to age discrimination. PG-ACIA therefore constructs a prior map from the query image to score spatial positions. A 3×3 Sobel operator is used to obtain a fine-scale response $E_{\mathrm{fine}}$, and the same operator is applied to a 5×5 Gaussian-blurred version of the image to obtain a coarse-scale response $E_{\mathrm{coarse}}$. The prior map is computed as

(2) $$P=E_{\mathrm{fine}}-\lambda E_{\mathrm{coarse}}$$

where λ denotes the prior coefficient. Based on the sensitivity analysis in Section 4.5.6, the default value is set to 0.4 as it suppresses coarse structural responses while preserving fine wrinkle cues. For each stage, *P* is resized to the corresponding feature resolution by bilinear interpolation. Informative positions are then selected row-wise and column-wise according to *P*. A base sparsity ratio of 0.15 is also selected based on sensitivity experiments so that interaction focuses on the most informative age-sensitive locations while maintaining sufficient spatial coverage. In implementation, the selected number is further adjusted according to the entropy of the prior map, and at least two positions are kept at coarse resolutions.

The second improvement is row–column axial decomposition. For each row *i*, the top-*K* column positions indicated by *P* are gathered from all *S* images and attention is applied along the sequence dimension to produce $X_{\mathrm{row}}$; the column branch is defined analogously to produce $X_{\mathrm{col}}$. This decomposition enables PG-ACIA to model direction-aware cross-image relations along horizontal and vertical axes. This directional design is consistent with the way many facial aging cues extend along horizontal or vertical directions, such as forehead lines and nasolabial folds. For locations outside the selected sparse set, a lightweight point branch performs ICA at a reduced hidden dimension of C/8 to produce $X_{\mathrm{point}}$. The three outputs are fused as

(3) $$X_{\mathrm{out}}=\alpha \left(X_{\mathrm{row}}+X_{\mathrm{col}}\right)+\beta X_{\mathrm{point}}$$

where α and β are learnable weights. They are initialized to 0.8 and 0.2, respectively, so that sparse axial interaction dominates the fusion while the point branch provides supplementary cross-image cues for unselected positions. In this way, PG-ACIA concentrates full-dimensional cross-image interaction on prior-selected locations, while the point branch supplies complementary cues for the remaining regions.

### 3.4. Anchored Regression Network

**Figure 6:**
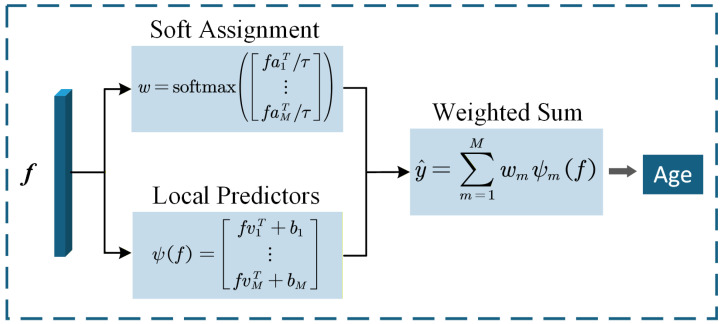
Illustration of the ARN module.

As illustrated in Figure 6, ARN predicts age through two parallel branches. The upper branch performs soft assignment by measuring the similarities between the query feature vector *f* and the *M* anchor vectors and then converts them into soft weights. The lower branch serves as local predictors by feeding *f* into *M* local linear regressors to produce *M* candidate age estimates. The final age is obtained by the weighted sum of these local predictions. In the final configuration, dot-product similarity and a learnable temperature are adopted. This allows ARN to assign larger weights to the most relevant anchors while keeping the prediction flexible when several anchors are similarly plausible. As a result, the final estimate becomes less sensitive to large appearance variation among same-age faces.

The initialization of anchors follows a boundary-aware two-step strategy. All *M* anchor vectors are initialized from training-sample features extracted by the initialized network, ensuring that they lie in the same feature space as *f*. First, each age class contributes one centroid anchor, computed as the mean feature of that class. Second, the remaining anchor budget is distributed across age classes as evenly as possible and each class selects its additional anchors from boundary candidates. These centroid and boundary-aware anchors are used to initialize the anchor bank. During training, the anchor vectors are further updated dynamically together with the backbone, allowing the anchors to adapt as the feature space evolves.

Boundary candidates are defined as follows. A sample is treated as a boundary candidate if it is farther from its own class centroid than from at least one adjacent-age centroid. Such samples are more likely to lie near age-transition regions. These candidates are ranked by their degree of boundary ambiguity, and the top-ranked ones are selected until the class quota is filled. If a class does not have enough boundary candidates, the remaining slots are filled with samples farthest from its centroid. In this way, the anchor set covers both representative and transitional age patterns. This design also preserves additional anchor coverage for sparse and long-tailed age regions rather than relying only on class centroids. ARN can therefore combine the most relevant local regressors more flexibly, making the final prediction less sensitive to large appearance variation among same-age faces and to imperfect reference cues.

## 4. Experiments

Experiments are conducted on four benchmark datasets that differ in scale, demographic composition, acquisition conditions and annotation granularity, namely MORPH Album II [30], MegaAge-Asian [31], FG-NET [32] and Adience [33]. For fair comparison, the results of competing methods are quoted directly from their original publications under the same evaluation protocols.

### 4.1. Datasets

MORPH Album II [30] is the most widely used benchmark in facial age estimation, containing 55,608 facial images from 13,617 subjects aged 16 to 77 years. The subjects cover multiple ethnic groups, including African-American, European-American and Hispanic individuals. The images are captured in a controlled law-enforcement setting, resulting in high image quality and reliable age annotations. In this work, an 80/20 random split is adopted, with 44,486 images for training and 11,122 for testing.

MegaAge-Asian [31] is a large-scale benchmark for facial age estimation on Asian faces. Under the official partition used in this work, it provides 40,000 training images and 3945 test images covering ages from 0 to 70 years. The images are collected under unconstrained conditions and exhibit substantial variations in illumination, pose and expression. Compared with MORPH II, MegaAge-Asian focuses on in-the-wild Asian faces and therefore complements the evaluation across demographic and acquisition settings.

FG-NET [32] contains 1002 facial images from 82 subjects aged 0 to 69 years. It includes longitudinal photographs of the same subject at multiple ages, together with noticeable variations in illumination and expression. Its limited scale and leave-one-person-out protocol make model training and evaluation on this benchmark particularly difficult. The leave-one-person-out (LOPO) protocol is adopted for evaluation.

Adience [33] is a real-world benchmark of unconstrained facial images collected from online photo albums. The original dataset contains 26,580 facial images annotated with eight discrete age groups (0–2, 4–6, 8–13, 15–20, 25–32, 38–43, 48–53, and 60+). Unlike MORPH II and MegaAge-Asian, it provides coarse group-level labels rather than exact ages. It also exhibits large variations in pose, illumination and occlusion, making it the most challenging in-the-wild benchmark among the four datasets. The standard 5-fold cross-validation protocol is followed, and the midpoint of each age group is used as the regression target.

### 4.2. Evaluation Metrics

For MORPH II, MegaAge-Asian and FG-NET, two standard metrics are used, namely mean absolute error (MAE) and cumulative score (CS). MAE is defined as

(4) $$\mathrm{MAE}=\frac{1}{N}\sum_{i=1}^{N}\left|y_{i}-{\hat{y}}_{i}\right|$$

where *N* denotes the number of test samples, and $y_{i}$ and ${\hat{y}}_{i}$ denote the ground-truth and predicted ages, respectively. A lower MAE indicates better estimation accuracy.

CS at threshold *x* is defined as

(5) $$\mathrm{CS}\left(x\right)=\frac{N_{e\leq x}}{N}\times 100\%$$

where *e* denotes the absolute error between the predicted age and the ground-truth age, *x* is the error threshold and $N_{e\leq x}$ denotes the number of test samples whose absolute error does not exceed *x*. Following common practice, CS(5) is reported in the experiments.

For Adience, MAE and accuracy (ACC) are reported. MAE is still computed using Equation (4). Since Adience provides age-group labels rather than exact ages, $y_{i}$ and ${\hat{y}}_{i}$ denote the ground-truth and predicted age-group indices, with the prediction assigned to the nearest age-group center. Therefore, the MAE on Adience reflects the average absolute error in age-group units rather than in years.

ACC is defined as

(6) $$\mathrm{ACC}=\frac{N_{\mathrm{correct}}}{N}\times 100\%$$

where $N_{\mathrm{correct}}$ denotes the number of test samples whose predicted age group matches the ground-truth age group. For Adience, a lower MAE and a higher ACC indicate better performance.

### 4.3. Implementation Details

All experiments are conducted on a server equipped with an NVIDIA GeForce RTX 5090 GPU (NVIDIA Corporation, Santa Clara, CA, USA) with 32 GB memory. Faces are detected and aligned using MediaPipe Face Mesh (Google LLC, Mountain View, CA, USA). They are then resized to 224×224 pixels and normalized with the ImageNet mean and standard deviation. During training, data augmentation includes random horizontal flipping, random rotation ($\pm 10^{\circ }$) and color jitter. No augmentation is applied at test time.

The model is trained with a batch size of 32 for up to 200 epochs with early stopping. The Adam optimizer is used with a base learning rate of $1\times 10^{-5}$ and a weight decay of $1\times 10^{-4}$. The learning rate is linearly warmed up for 5 epochs and then decayed with cosine annealing to 0.1 times the initial value.

For each query image, eight reference images are sampled from the training set during both training and testing. For MORPH II, MegaAge-Asian and FG-NET, the references share the same age label as the query image. For Adience, they are sampled from the same age group. Together with the query image, they form an input sequence of length 9. For FG-NET, the sequence length is reduced to 4 and the age-matching tolerance is relaxed to ±1 year to accommodate the limited dataset size.

### 4.4. Comparative Evaluation

#### 4.4.1. Quantitative Results on MORPH II

We compare the CIIT with some baselines and SOTA methods on MORPH II, as shown in Table 1. Overall, the CIIT achieves highly competitive performance, with an MAE of 1.19 and the best reported CS(5) of 99.58%. Among recent single-image-based methods, HR [34] and GLAE [35] report slightly lower MAEs of 1.13 and 1.14, respectively. Their advantages may be related to stronger age-specific regression design in HR and stronger long-tailed age modeling together with larger-scale pretraining in GLAE. Nevertheless, the CIIT provides the highest reported CS(5), indicating stronger tolerance-based accuracy. Among methods that further introduce relation modeling, such as TAA-GCN [36] and OrdCon [25], the CIIT achieves lower MAE than both and also provides the highest reported CS(5). These results demonstrate the strong competitiveness of the CIIT on the large-scale MORPH II benchmark.

**Figure 7:**
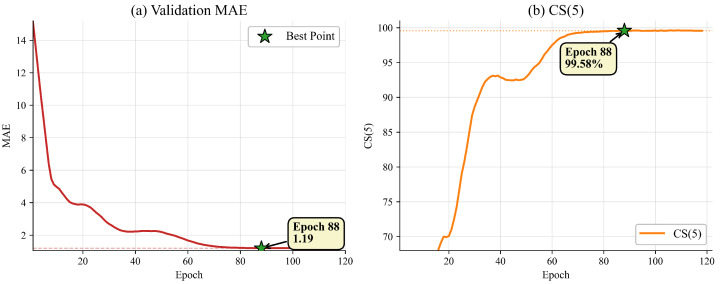
Validation MAE and CS(5) convergence curves on MORPH II. The red dashed line in subfigure (**a**) marks the epoch with the best validation MAE.

In addition to the quantitative results in Table 1, Figure 7 shows the stable convergence behavior of the CIIT on MORPH II. The validation MAE drops rapidly in the early epochs and then decreases more gradually until reaching 1.19 at epoch 88. Meanwhile, CS(5) rises quickly at the beginning and then stabilizes at 99.58%, with no evident late-stage degradation.

#### 4.4.2. Quantitative Results on MegaAge-Asian

We compare the CIIT with representative and SOTA single-image-based methods on MegaAge-Asian, as shown in Table 2. Overall, the CIIT achieves the best MAE of 1.25 and the best CS(5) of 98.76%. Among the compared methods, the best previous CS(5) is 87.24% from PVP+VGG16 [45], while the best previous MAE is 2.93 from DAA [13]. Compared with these strongest prior results, the CIIT improves CS(5) by 11.52% and reduces MAE by 1.68. Notably, PVP+VGG16 relies on ImageNet, IMDB-WIKI and AFAD pretraining, whereas the CIIT uses only ImageNet pretraining. Since all competing methods in Table 2 estimate age from a single image, whereas the CIIT is the only method here based on a cross-identity image sequence, these results support the effectiveness of the proposed multi-image modeling strategy on MegaAge-Asian.

#### 4.4.3. Quantitative Results on FG-NET

To further assess the CIIT under the challenging LOPO setting, we compare it with representative and SOTA methods on the small-scale FG-NET benchmark, as shown in Table 3. The CIIT achieves the best MAE of 1.42 and the best CS(5) of 95.4%. Among recent single-image-based methods, PML [50] reports the strongest previous MAE of 2.16. LRA-GNN [27] is also a relevant comparator because, like PG-ACIA in the CIIT, it goes beyond global facial representation by explicitly modeling structured spatial dependencies. However, LRA-GNN does this within a single image through keypoint-based graph reasoning, whereas the CIIT learns shared age cues across a cross-identity reference sequence. The CIIT improves over LRA-GNN from 2.14 to 1.42 in MAE and from 91.6% to 95.4% in CS(5). These results show that the CIIT remains highly effective on the small-scale FG-NET benchmark.

#### 4.4.4. Quantitative Results on Adience

To examine whether the CIIT remains effective under coarse age-group supervision, we compare it with representative and SOTA methods on Adience, as shown in Table 4. Among methods reporting both MAE and ACC, the CIIT achieves the best overall performance, with an MAE of 0.24 and an ACC of 69.9%. Compared with the best previous results within this group, the CIIT reduces MAE by 0.12 and improves ACC by 3.7%. Agbo-Ajala CNN [51] and ViT-hSeq [52] are the only two methods that report higher ACC than the CIIT. Both are tailored specifically to age-group classification on Adience. Their advantage is therefore limited to the classification metric rather than the overall two-metric comparison. Overall, these results show that the CIIT remains competitive under coarse age-group supervision.

### 4.5. Ablation Study

Six ablation studies are conducted on MORPH II to examine the main design choices regarding the CIIT. MAE and CS(5) are reported throughout unless otherwise stated. These analyses cover component-level ablation, PG-ACIA design, reference sequence length, regression head design, the number of anchors and hyperparameter sensitivity.

#### 4.5.1. Component-Level Ablation

Table 5 compares the full model with three removal variants and one variant replacing sparse ICA with dense ICA. A2, the variant without cross-image attention, causes the largest performance degradation, with MAE increasing by 0.39. This shows that cross-identity interaction is essential because same-age faces still vary markedly across identities. A1, obtained by removing CSE, causes the second-largest increase, confirming its role in capturing multi-scale aging cues. In A4, removing ARN raises MAE from 1.19 to 1.34, showing that soft anchor aggregation with a learnable temperature is more effective than a plain global regression head. In A3, replacing PG-ACIA with dense ICA raises MAE to 1.28, indicating that prior-guided sparse selection provides an additional gain beyond dense axial attention.

#### 4.5.2. Analysis of PG-ACIA Design

Table 6 presents six progressive PG-ACIA settings from P1 to P6. P1 starts without cross-image interaction and gives an MAE of 1.58. Adding dense global cross-image attention in P2 reduces it to 1.52, showing the value of cross-identity comparison. P3 then adds axial decomposition and gives the largest single-step gain, lowering MAE to 1.28. This suggests that row–column axial decomposition provides a more suitable structured interaction pattern for cross-image alignment, which is consistent with the directional structure of many facial aging cues. P4 adds a single-scale Sobel prior, but MAE rises to 1.33, indicating that coarse gradients from hairlines and facial shadows can mislead attention. Replacing it with the multi-scale differential prior in P5 lowers MAE to 1.28. P6 further adds top-*k* sparsification and reduces MAE to 1.19. These results show that both prior quality and sparse selection are important to the final PG-ACIA design.

#### 4.5.3. Effect of Reference Sequence Length

**Figure 8:**
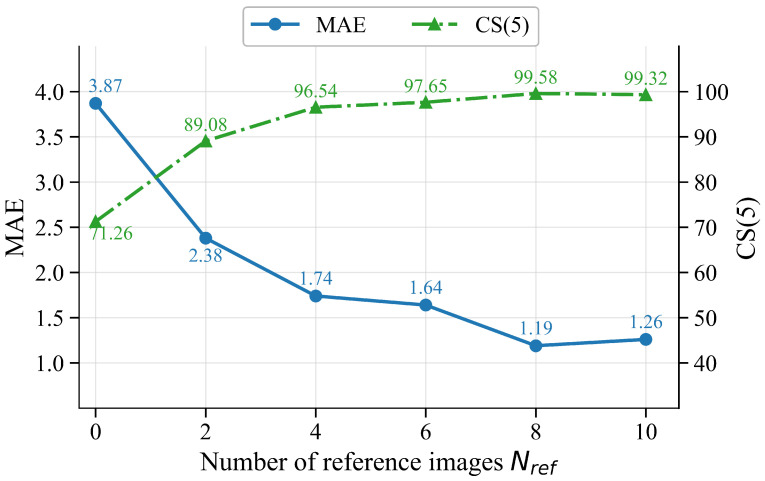
Effect of reference sequence length $N_{\mathrm{ref}}$ on MAE and CS(5) on MORPH II.

Table 7 compares six reference sequence lengths $N_{\mathrm{ref}}\in \left\{0,2,4,6,8,10\right\}$ on MORPH II. In S0, using no reference images gives an MAE of 3.87 and a CS(5) of 71.26. When two reference images are introduced in S1, the MAE drops by 1.49 to 2.38 and CS(5) rises to 89.08, directly showing the benefit of reference images for cross-identity comparison. As $N_{\mathrm{ref}}$ increases from S1 to S4, the performance continues to improve and S4 achieves the best MAE of 1.19 and CS(5) of 99.58. When the sequence length is further increased to 10 in S5, the MAE rises to 1.26 and CS(5) drops slightly to 99.32. A likely reason is that, for some ages, it becomes harder to sample sufficiently diverse same-age reference images. The additional references then become more repetitive and contribute less complementary age information. This trend is also consistent with Figure 8, where the MAE drops steeply from $N_{\mathrm{ref}}\;=\;0$ to 4 and then flattens, whereas the CS(5) curve rises rapidly and nearly saturates at $N_{\mathrm{ref}}\;=\;8$. Longer reference sequences also increase computation and GPU memory demand. Therefore, $N_{\mathrm{ref}}\;=\;8$ is adopted as the default setting because it gives the best accuracy with a moderate sequence length.

#### 4.5.4. Analysis of Regression Head Design

**Figure 9:**
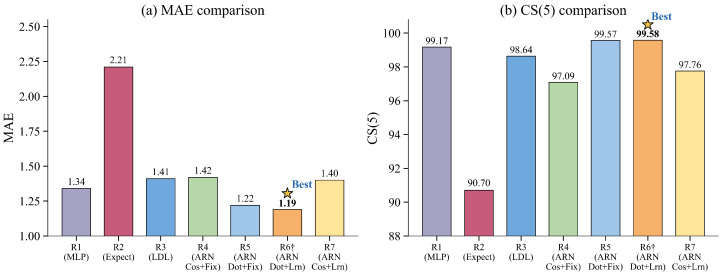
Performance comparison across seven regression head configurations on MORPH II. The optimal configuration is marked with ★.

Table 8 compares three non-anchor regression heads, namely MLP, expectation regression and LDL, together with four ARN variants, R4 to R7, under different similarity functions and temperature strategies. Among all the compared heads, R6 achieves the best overall performance, with an MAE of 1.19 and a CS(5) of 99.58%. In contrast, the three non-anchor baselines, namely MLP, expectation regression and LDL, yield MAEs of 1.34, 2.21 and 1.41, respectively. These results show that the anchor-based soft-assignment design is more effective than conventional regression heads when the design is properly configured. First, to compare similarity functions under the same temperature strategy, R5 outperforms R4 under fixed temperature, and R6 also outperforms R7 under learnable temperature. These results consistently show that dot-product similarity is more suitable than cosine similarity for anchor weighting. Second, to compare temperature strategies under the same similarity function, R6 further improves over R5 in the dot-product setting, while R7 slightly improves over R4 in the cosine setting. This indicates that learnable temperature is beneficial in both similarity families, with a more noticeable gain under dot-product similarity. These two trends are also evident in Figure 9. A possible reason is that dot product preserves both direction and magnitude information, which may help ARN to better match anchors to the shared age cues learned through cross-identity interaction. Learnable temperature then adjusts the sharpness of anchor weighting, making the final prediction more robust when reference cues are less reliable.

#### 4.5.5. Effect of the Number of Anchors in ARN

**Figure 10:**
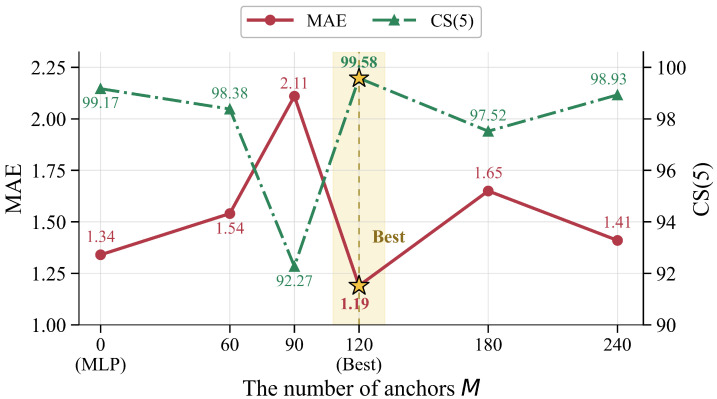
Effect of the number of anchors *M* on MAE and CS(5) on MORPH II. The optimal configuration (M=120) is marked with ★.

Table 9 compares six settings of the number of anchors on MORPH II, with *M* set to 0, 60, 90, 120, 180 and 240. N0 uses no anchors and corresponds to the MLP baseline. With M=60, N1 essentially uses centroid anchors for each age class yet still performs worse than N0, suggesting that this setting is too coarse to cover age variation. N2 gives the weakest performance because M=90 is not well aligned with the roughly 60 age classes in MORPH II, leading to inconsistent anchor granularity. N3 achieves the best result at M=120, with an MAE of 1.19 and a CS(5) of 99.58. In this setting, each age is represented not only by a centroid anchor but also by an additional boundary-oriented anchor, which better balances representative and transitional age patterns. When the number of anchors is further increased, N4 and N5 yield MAEs of 1.65 and 1.41, both worse than N3. A possible reason is that an overly fine-grained anchor set scatters the shared facial characteristics learned through cross-identity interaction across nearby anchors, making anchor weighting less concentrated and the final prediction less stable. Figure 10 shows the same non-monotonic trend, with performance worst at M=90, best at M=120, and lower again for larger values.

#### 4.5.6. Sensitivity to Prior Coefficient and Sparsity Ratio

**Figure 11:**
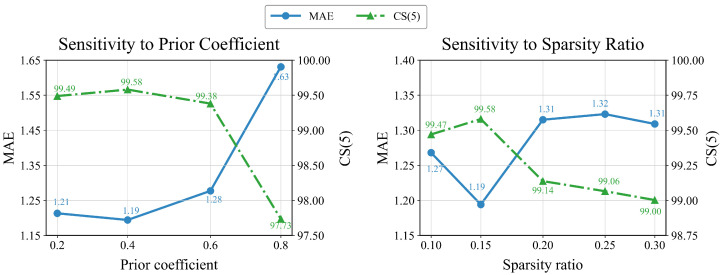
Sensitivity analysis of the prior coefficient and sparsity ratio on MORPH II. The selected setting (0.4,0.15) yields the best overall performance in the two studies.

Table 10 reports the sensitivity of the prior coefficient and the sparsity ratio on MORPH II. For the prior coefficient in Equation (2), the default value of 0.4 gives the best overall result, whereas both smaller and larger values lead to worse performance. This suggests that too weak a coefficient cannot suppress distracting coarse gradients sufficiently, while too large a coefficient also removes useful low-frequency facial structure. A similar trend is observed for the sparsity ratio. The default value of 0.15 again gives the best overall result, while neighboring settings remain close but consistently inferior. A likely reason is that an overly small ratio discards informative positions, whereas a larger ratio introduces more low-priority responses and weakens the benefit of sparsification. Therefore, the final model adopts 0.4 as the prior coefficient and 0.15 as the sparsity ratio because they provide the best balance under the current protocol. These trends are also reflected in Figure 11.

### 4.6. Additional Evaluation

Beyond the ablation study, we further evaluate the CIIT from three complementary perspectives, namely inference efficiency, age-range robustness and representative visual results.

#### 4.6.1. Inference Efficiency and Deployment Discussion

To assess the practical deployment cost of the CIIT, we benchmark the five component-level variants under the same input setting. We report parameter count to reflect model size, FLOPs to measure theoretical computational cost, latency per query and throughput in query/s to characterize inference speed, and peak inference memory to indicate deployment-time memory usage. Here, query/s is the FPS-equivalent metric in our sequence-based setting because one query corresponds to one nine-image input sequence.

Table 11 shows that the full model A0 achieves 9.95 ms per query and 100.52 queries/s, indicating practical deployment on a modern GPU. A2 is the fastest because removing cross-image interaction simplifies computation, whereas A3 requires the largest FLOPs and peak inference memory, showing the extra deployment cost of dense ICA relative to the prior-guided sparse design. Although A4 removes ARN, it is still slower than A0, suggesting that the main efficiency advantage of the CIIT comes from sparse cross-image interaction rather than from simplifying the regression head. Overall, the final CIIT configuration provides a favorable balance between accuracy and efficiency, and its online cost can be further reduced by precomputing reference features offline.

#### 4.6.2. Age-Range Evaluation

**Figure 12:**
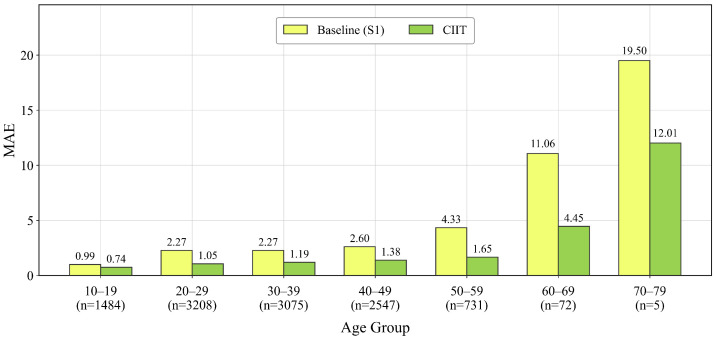
Age-range MAE comparison between the short-sequence baseline S1 ($N_{\mathrm{ref}}\;=\;2$) and the full CIIT model on MORPH II. The test set is divided into 10-year age groups, and the number below each group denotes the number of test samples.

To further examine the robustness of the CIIT across different age ranges, we evaluate MAE on MORPH II by dividing the test set into 10-year age groups, as shown in Figure 12. For this analysis, we compare the full CIIT model with the short-sequence baseline S1 ($N_{\mathrm{ref}}\;=\;2$). This comparison is particularly interpretable because S1 shares the same backbone, training protocol and label-based reference construction strategy with the full model while differing mainly in reference sequence length. The grouped results therefore directly reflect how richer cross-identity reference interaction affects performance across age ranges.

Figure 12 shows that the CIIT achieves lower MAE than S1 in all the populated age groups. The improvement is especially clear from 20–59 years, and it remains evident in the 60–69 and 70–79 groups despite the much smaller number of test samples. The error increases in the two oldest groups for both models, which is likely due to the severe sparsity of high-age samples in MORPH II. These results demonstrate that the proposed model maintains a consistent advantage across different age ranges and thus supports its robustness beyond the overall average metric.

#### 4.6.3. Visualization of Estimation Results

**Figure 13:**
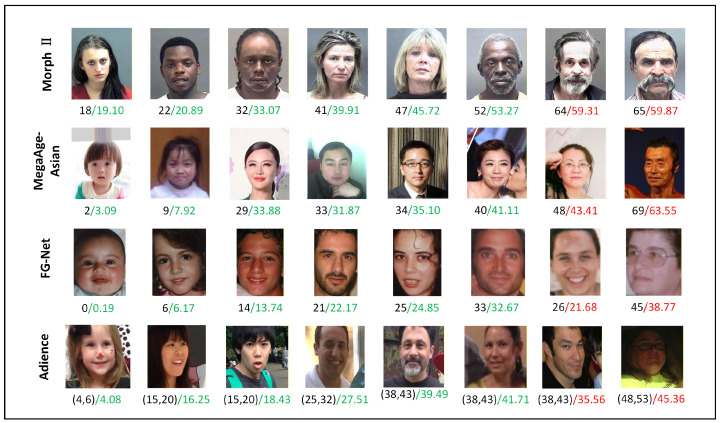
Prediction examples from all four benchmarks. Black numbers denote ground-truth labels, and green and red numbers denote accurate and erroneous predictions, respectively. Adience labels denote age-group intervals, and a prediction is regarded as accurate if it falls within the interval.

Representative results are shown in Figure 13. On MORPH II and MegaAge-Asian, the CIIT predicts children, young adults and middle-aged faces more accurately than elderly faces. The oldest examples are consistently underestimated, which likely reflects the long-tail scarcity of elderly samples in these datasets. With fewer age-matched reference images available at older ages, reference construction becomes less reliable, and the benefit of cross-identity interaction is correspondingly reduced. The FG-NET row remains accurate in most cases despite the limited dataset size, although the 26- and 45-year-old examples are underestimated. On Adience, most predictions stay within the annotated age interval, while the two errors both fall below the target interval. Overall, these examples suggest that the CIIT is more reliable when informative age-matched references are available, whereas long-tail ages and coarse age-group supervision remain more challenging.

## 5. Conclusions

This paper presented the CIIT for facial age estimation. Unlike single-image-based methods, the CIIT estimates age through interaction within a cross-identity image sequence formed by one query face and same-age reference faces. By combining CSE with PG-ACIA, the CIIT captures both fine texture changes and coarse structural cues while aligning age-sensitive features across different identities. This design helps the model to focus on shared age-related characteristics and reduces interference from identity-specific appearance variation. Experiments on MORPH II, MegaAge-Asian, FG-NET and Adience showed that the CIIT achieves competitive performance across different evaluation settings. The ablation studies further verified the effectiveness of its key design choices. The current framework still depends on age labels to construct reference sequences and requires joint inference over multiple images. Future work will therefore explore label-free reference construction and lighter interaction designs for practical deployment.

## Figures and Tables

**Table 1:** Comparison with state-of-the-art methods on MORPH II. The best result in each column is shown in bold. − indicates that the metric was not reported in the original work.

Method	Year	Pretrained	MAE	CS(5)
DLDL-V2 [18]	2018	MS-Celeb-1M	1.97	−
DEX [3]	2018	IMDB-WIKI	2.68	78.10
DHAA [37]	2019	ImageNet	1.91	−
MWR [23]	2022	IMDB-WIKI	2.00	95.00
GOL [38]	2022	ImageNet	2.09	94.20
MetaAge [39]	2022	IMDB-WIKI	1.81	−
GLAE [35]	2023	MS-Celeb-1M	1.14	−
DAA [13]	2023	IMDB-WIKI	2.06	−
OLDL [21]	2023	ImageNet	2.45	−
TAA-GCN [36]	2023	−	1.69	−
HR [34]	2023	ImageNet	**1.13**	−
MSDNN [40]	2024	ImageNet	2.59	86.66
GroupFace [26]	2024	IMDB-WIKI	1.86	−
ConvNeXt-Trans [41]	2025	ImageNet	2.26	−
CSCS-Swin [16]	2025	ImageNet	2.26	−
MGP-Net [42]	2025	IMDB-WIKI	2.25	91.82
MCGRL [43]	2025	−	2.39	89.90
OrdCon [25]	2025	−	2.21	−
LRA-GNN [27]	2025	−	1.79	−
SA-LDL [44]	2025	−	1.75	92.20
GFL [29]	2025	IMDB-WIKI	1.57	−
**Ours**	2026	ImageNet	1.19	**99.58**

**Table 2:** Comparison with state-of-the-art methods on MegaAge-Asian. The best result in each column is shown in bold. − indicates that the metric was not reported in the original work.

Method	Year	Pretrained	MAE	CS(5)
Posterior [31]	2017	MS-Celeb-1M	−	82.15
SSR-Net [5]	2018	IMDB-WIKI	−	74.10
LRN [46]	2020	IMDB-WIKI	−	82.95
VGG+Distillation [45]	2020	ImageNet, IMDB-WIKI, AFAD	−	83.01
PVP+VGG16 [45]	2020	ImageNet, IMDB-WIKI, AFAD	−	87.24
DAA [13]	2023	−	2.93	84.89
ALD-Net [47]	2023	−	−	81.20
TDT [48]	2024	ImageNet	−	85.42
SA-Hierarchical [49]	2025	ImageNet	3.09	82.30
**Ours**	2026	ImageNet	**1.25**	**98.76**

**Table 3:** Comparison with state-of-the-art methods on FG-NET under the LOPO protocol. The best result in each column is shown in bold. − indicates that the metric was not reported in the original work.

Method	Year	Pretrained	MAE	CS(5)
MV-Loss [19]	2018	ImageNet, IMDB-WIKI	2.68	−
BridgeNet [24]	2019	ImageNet, IMDB-WIKI	2.56	86.0
AVDL [20]	2020	IMDB-WIKI	2.32	−
PML [50]	2021	IMDB-WIKI	2.16	−
MWR [23]	2022	IMDB-WIKI	2.23	91.1
DAA [13]	2023	IMDB-WIKI	2.19	−
MCGRL [43]	2025	−	2.86	88.0
OrdCon [25]	2025	−	2.85	−
MGP-Net [42]	2025	IMDB-WIKI	2.28	90.3
LRA-GNN [27]	2025	−	2.14	91.6
**Ours**	2026	ImageNet	**1.42**	**95.4**

**Table 4:** Comparison with state-of-the-art methods on Adience. The best result in each column is shown in bold. − indicates that the metric was not reported in the original work.

Method	Year	Pretrained	MAE	ACC
SORD [53]	2019	ImageNet	0.49	59.6
AL-ResNet-34 [54]	2019	ImageNet, IMDB-WIKI	−	67.5
Agbo-Ajala CNN [51]	2020	IMDB-WIKI	−	83.1
EfficientNet-B4 [55]	2021	ImageNet	−	81.1
OrdinalCLIP [56]	2022	CLIP	0.47	61.2
MWR [23]	2022	ImageNet	0.45	62.6
GOL [38]	2022	ImageNet	0.43	62.5
CIG-PVT [57]	2023	ImageNet	0.43	64.4
L2RCLIP [58]	2023	CLIP	0.36	66.2
MiVOLO-D1 [28]	2023	LAGENDA	−	68.7
ViT-hSeq [52]	2024	ImageNet	−	**84.9**
SCG-Net [59]	2025	LAGENDA	0.36	65.2
**Ours**	2026	ImageNet	**0.24**	69.9

**Table 5:** Component-level ablation on MORPH II. The best result in each column is shown in bold. ✓ and × indicate that the corresponding component is enabled and removed, respectively. Dense denotes replacing sparse ICA with dense ICA. The ΔMAE column reports the MAE difference from the full model, and − marks the reference row.

ID	Configuration	CSE	ICA	ARN	MAE	CS(5)	ΔMAE
A0	Full model	✓	✓	✓	**1.19**	**99.58**	−
A1	w/o CSE	×	✓	✓	1.43	98.91	+0.24
A2	w/o ICA	✓	×	✓	1.58	97.86	+0.39
A3	Dense ICA	✓	Dense	✓	1.28	99.39	+0.09
A4	w/o ARN	✓	✓	×	1.34	99.17	+0.15

**Table 6:** Progressive design analysis of PG-ACIA on MORPH II. The best result in each column is shown in bold. ✓ and × indicate that the corresponding operation is enabled and disabled, respectively. − indicates not applicable. The ΔMAE column reports the MAE change from the previous row, and − in the ΔMAE column marks the reference row.

ID	Cross-img	Axial	Prior	Prior Type	Sparse	MAE	CS(5)	ΔMAE
P1	×	−	−	−	−	1.58	97.86	−
P2	✓	×	×	−	−	1.52	98.31	−0.06
P3	✓	✓	×	−	−	1.28	99.39	−0.24
P4	✓	✓	✓	Single-scale	×	1.33	99.26	+0.05
P5	✓	✓	✓	Multi-scale	×	1.28	99.39	−0.05
P6	✓	✓	✓	Multi-scale	✓	**1.19**	**99.58**	−0.09

**Table 7:** Effect of reference sequence length on MORPH II. The best result in each column is shown in bold. The ΔMAE column reports the MAE change from the previous row. − marks the reference row.

ID	$N_{\mathrm{ref}}$	MAE	CS(5)	ΔMAE
S0	0	3.87	71.26	−
S1	2	2.38	89.08	−1.49
S2	4	1.74	96.54	−0.64
S3	6	1.64	97.65	−0.10
S4	8	**1.19**	**99.58**	−0.45
S5	10	1.26	99.32	+0.07

**Table 8:** Analysis of regression head design on MORPH II. The best result in each column is shown in bold. N/A indicates that anchor similarity and temperature scaling are not used in the MLP, expectation regression or LDL heads. The ΔMAE column reports the MAE change from the previous row. − marks the reference row.

ID	Reg. Head	Similarity	Temperature	MAE	CS(5)	ΔMAE
R1	MLP	N/A	N/A	1.34	99.17	−
R2	Expectation Regression	N/A	N/A	2.21	90.70	+0.87
R3	LDL	N/A	N/A	1.41	98.64	−0.80
R4	ARN	Cosine	Fixed τ=1.0	1.42	97.09	+0.01
R5	ARN	Dot	Fixed τ=1.0	1.22	99.57	−0.20
R6	ARN	Dot	Learnable	**1.19**	**99.58**	−0.03
R7	ARN	Cosine	Learnable	1.40	97.76	+0.21

**Table 9:** Effect of the number of anchors *M* on MORPH II. The best result in each column is shown in bold. The ΔMAE column reports the MAE change from the previous row. − marks the reference row.

ID	*M*	MAE	CS(5)	ΔMAE
N0	0	1.34	99.17	−
N1	60	1.54	98.38	+0.20
N2	90	2.11	92.27	+0.57
N3	120	**1.19**	**99.58**	−0.92
N4	180	1.65	97.52	+0.46
N5	240	1.41	98.93	−0.24

**Table 10:** Sensitivity analysis of the prior coefficient and sparsity ratio on MORPH II. The best result in each group is shown in bold, and the ΔMAE columns report the MAE change from the previous row. − marks the reference row.

Prior Coefficient				Sparsity Ratio			
Value	MAE	CS(5)	ΔMAE	Value	MAE	CS(5)	ΔMAE
0.2	1.21	99.49	−	0.10	1.27	99.47	−
0.4	**1.19**	**99.58**	−0.02	0.15	**1.19**	**99.58**	−0.08
0.6	1.28	99.41	+0.09	0.20	1.32	99.17	+0.13
0.8	1.63	97.73	+0.35	0.25	1.32	99.07	0.00
				0.30	1.31	99.01	−0.01

**Table 11:** Inference efficiency comparison of the five component-level variants on MORPH II, measured on an NVIDIA GeForce RTX 5090 with synthetic input of size 9×3×224×224.

ID	Configuration	Params (M)	FLOPs (G)	Latency (ms/query)	Throughput (query/s)	Peak Mem (GB)
A0	Full model	33.07	101.25	9.95	100.52	0.26
A1	w/o CSE	31.86	76.09	11.84	84.49	0.26
A2	w/o ICA	28.21	107.44	7.00	142.84	0.24
A3	Dense ICA	33.07	134.47	10.89	91.81	0.59
A4	w/o ARN	33.18	101.70	12.94	77.29	0.26

## Data Availability

Data will be made available on request.

## References

[1] Ojala T., Pietikainen M., Maenpaa T. (2002). Multiresolution gray-scale and rotation invariant texture classification with local binary patterns. IEEE Trans. Pattern Anal. Mach. Intell..

[2] Daugman J.G. (1985). Uncertainty relation for resolution in space, spatial frequency, and orientation optimized by two-dimensional visual cortical filters. J. Opt. Soc. Am. A.

[3] Rothe R., Timofte R., Van Gool L. (2018). Deep expectation of real and apparent age from a single image without facial landmarks. Int. J. Comput. Vis..

[4] Chen S., Zhang C., Dong M., Le J., Rao M. Using ranking-CNN for age estimation. Proceedings of the IEEE Conference on Computer Vision and Pattern Recognition.

[5] Yang T.Y., Huang Y.H., Lin Y.Y., Hsiu P.C., Chuang Y.Y. Ssr-net: A compact soft stagewise regression network for age estimation. Proceedings of the IJCAI.

[6] Zhang C., Liu S., Xu X., Zhu C. C3AE: Exploring the limits of compact model for age estimation. Proceedings of the IEEE/CVF Conference on Computer Vision and Pattern Recognition.

[7] Lin T.Y., Dollár P., Girshick R., He K., Hariharan B., Belongie S. Feature pyramid networks for object detection. Proceedings of the IEEE Conference on Computer Vision and Pattern Recognition.

[8] Hu J., Shen L., Sun G. Squeeze-and-excitation networks. Proceedings of the IEEE Conference on Computer Vision and Pattern Recognition.

[9] Woo S., Park J., Lee J.Y., Kweon I.S. (2018). Cbam: Convolutional block attention module. Proceedings of the European Conference on Computer Vision (ECCV).

[10] Lin Y., Shen J., Wang Y., Pantic M. (2025). FP-Age: Leveraging Face Parsing Attention for Facial Age Estimation in the Wild. IEEE Trans. Image Process..

[11] Dosovitskiy A., Beyer L., Kolesnikov A., Weissenborn D., Zhai X., Unterthiner T., Dehghani M., Minderer M., Heigold G., Gelly S. (2020). An image is worth 16x16 words: Transformers for image recognition at scale. arXiv.

[12] Liu Z., Lin Y., Cao Y., Hu H., Wei Y., Zhang Z., Lin S., Guo B. Swin transformer: Hierarchical vision transformer using shifted windows. Proceedings of the IEEE/CVF International Conference on Computer Vision.

[13] Chen P., Zhang X., Li Y., Tao J., Xiao B., Wang B., Jiang Z. DAA: A Delta Age AdaIN operation for age estimation via binary code transformer. Proceedings of the IEEE/CVF Conference on Computer Vision and Pattern Recognition.

[14] Qin L., Wang M., Deng C., Wang K., Chen X., Hu J., Deng W. (2023). SwinFace: A multi-task transformer for face recognition, expression recognition, age estimation and attribute estimation. IEEE Trans. Circuits Syst. Video Technol..

[15] Tang H., Li Z., Zhang D., He S., Tang J. (2024). Divide-and-Conquer: Confluent Triple-Flow Network for RGB-T Salient Object Detection. IEEE Trans. Pattern Anal. Mach. Intell..

[16] Xu L., Hu C., Shu X., Yu H. (2025). Cross spatial and Cross-Scale Swin Transformer for fine-grained age estimation. Comput. Electr. Eng..

[17] Geng X., Yin C., Zhou Z.H. (2013). Facial age estimation by learning from label distributions. IEEE Trans. Pattern Anal. Mach. Intell..

[18] Gao B.B., Zhou H.Y., Wu J., Geng X. Age estimation using expectation of label distribution learning. Proceedings of the IJCAI’18: Proceedings of the 27th International Joint Conference on Artificial Intelligence.

[19] Pan H., Han H., Shan S., Chen X. Mean-variance loss for deep age estimation from a face. Proceedings of the IEEE Conference on Computer Vision and Pattern Recognition.

[20] Wen X., Li B., Guo H., Liu Z., Hu G., Tang M., Wang J. (2020). Adaptive variance based label distribution learning for facial age estimation. Proceedings of the European Conference on Computer Vision.

[21] Wen C., Zhang X., Yao X., Yang J. Ordinal label distribution learning. Proceedings of the IEEE/CVF International Conference on Computer Vision.

[22] Cao W., Mirjalili V., Raschka S. (2020). Rank consistent ordinal regression for neural networks with application to age estimation. Pattern Recognit. Lett..

[23] Shin N.H., Lee S.H., Kim C.S. Moving window regression: A novel approach to ordinal regression. Proceedings of the IEEE/CVF Conference on Computer Vision and Pattern Recognition.

[24] Li W., Lu J., Feng J., Xu C., Zhou J., Tian Q. Bridgenet: A continuity-aware probabilistic network for age estimation. Proceedings of the IEEE/CVF Conference on Computer Vision and Pattern Recognition.

[25] Wang H., Sanchez V., Li C.T., Clarke N. (2025). From Age Estimation to Age-Invariant Face Recognition: Generalized Age Feature Extraction Using Order-Enhanced Contrastive Learning. IEEE Trans. Inf. Forensics Secur..

[26] Zhang Y., Shou Y., Ai W., Meng T., Li K. (2024). GroupFace: Imbalanced age estimation based on multi-hop attention graph convolutional network and group-aware margin optimization. IEEE Trans. Inf. Forensics Secur..

[27] Zhang Y., Shou Y., Ai W., Meng T., Li K. (2025). LRA-GNN: Latent relation-aware graph neural network with initial and dynamic residual for facial age estimation. Expert Syst. Appl..

[28] Kuprashevich M., Tolstykh I. (2023). Mivolo: Multi-input transformer for age and gender estimation. Proceedings of the International Conference on Analysis of Images, Social Networks and Texts.

[29] Zhao Q., Li Y. (2025). Facial age estimation by group-centric feature learning. Expert Syst. Appl..

[30] Ricanek K., Tesafaye T. MORPH: A longitudinal image database of normal adult age-progression. Proceedings of the International Conference on Automatic Face and Gesture Recognition.

[31] Zhang Y., Liu L., Li C., Loy C.C. (2017). Quantifying facial age by posterior of age comparisons. Proceedings of the British Machine Vision Conference (BMVC).

[32] Panis G., Lanitis A., Tsapatsoulis N., Cootes T. (2016). Overview of research on facial ageing using the FG-NET ageing database. IET Biom..

[33] Eidinger E., Enbar R., Hassner T. (2014). Age and gender estimation of unfiltered faces. IEEE Trans. Inf. Forensics Secur..

[34] Hiba S., Keller Y. (2023). Hierarchical attention-based age estimation and bias analysis. IEEE Trans. Pattern Anal. Mach. Intell..

[35] Bao Z., Tan Z., Li J., Wan J., Ma X., Lei Z. (2023). General vs. long-tailed age estimation: An approach to kill two birds with one stone. IEEE Trans. Image Process..

[36] Korban M., Youngs P., Acton S.T. (2023). Taa-gcn: A temporally aware adaptive graph convolutional network for age estimation. Pattern Recognit..

[37] Tan Z., Yang Y., Wan J., Guo G., Li S.Z. Deeply-learned Hybrid Representations for Facial Age Estimation. Proceedings of the IJCAI’19: Proceedings of the 28th International Joint Conference on Artificial Intelligence.

[38] Lee S.H., Shin N.H., Kim C.S. (2022). Geometric order learning for rank estimation. Adv. Neural Inf. Process. Syst..

[39] Li W., Lu J., Wuerkaixi A., Feng J., Zhou J. (2022). MetaAge: Meta-learning personalized age estimators. IEEE Trans. Image Process..

[40] Bekhouche S.E., Benlamoudi A., Dornaika F., Telli H., Bounab Y. (2024). Facial age estimation using multi-stage deep neural networks. Electronics.

[41] Maroun G., Bekhouche S.E., Charafeddine J., Dornaika F. (2025). Integrating ConvNeXt and vision transformers for enhancing facial age estimation. Comput. Vis. Image Underst..

[42] Zang H.X., Xiao Q. (2025). Multi-grained pooling network for age estimation in degraded low-resolution images. Sci. Rep..

[43] Shou Y., Cao X., Liu H., Meng D. (2025). Masked contrastive graph representation learning for age estimation. Pattern Recognit..

[44] Wu B., Ai Z., Jiang J., Zhu C., Xu S. (2025). Stage-wise Adaptive Label Distribution for Facial Age Estimation. arXiv.

[45] Zhao Q., Dong J., Yu H., Chen S. (2020). Distilling ordinal relation and dark knowledge for facial age estimation. IEEE Trans. Neural Netw. Learn. Syst..

[46] Li P., Hu Y., Wu X., He R., Sun Z. (2020). Deep label refinement for age estimation. Pattern Recognit..

[47] He J., Hu C., Wang L. (2023). Facial age estimation based on asymmetrical label distribution. Multimed. Syst..

[48] Chen P., Zhang X., Zhou C., Fan D., Tu P., Zhang L., Qian Y. Learning triangular distribution in visual world. Proceedings of the IEEE/CVF Conference on Computer Vision and Pattern Recognition.

[49] Qiao X., Hu C., Qin B. (2025). Semantic Attention Guided Hierarchical Decision Network for Age Estimation. Appl. Sci..

[50] Deng Z., Liu H., Wang Y., Wang C., Yu Z., Sun X. Pml: Progressive margin loss for long-tailed age classification. Proceedings of the IEEE/CVF Conference on Computer Vision and Pattern Recognition.

[51] Agbo-Ajala O., Viriri S. (2020). Deeply learned classifiers for age and gender predictions of unfiltered faces. Sci. World J..

[52] Singh A., Singh V.K. (2024). A hybrid transformer–sequencer approach for age and gender classification from in-wild facial images. Neural Comput. Appl..

[53] Diaz R., Marathe A. Soft labels for ordinal regression. Proceedings of the IEEE/CVF Conference on Computer Vision and Pattern Recognition.

[54] Zhang K., Liu N., Yuan X., Guo X., Gao C., Zhao Z., Ma Z. (2019). Fine-grained age estimation in the wild with attention LSTM networks. IEEE Trans. Circuits Syst. Video Technol..

[55] Aruleba I., Viriri S. (2021). Deep learning for age estimation using EfficientNet. Proceedings of the International Work-Conference on Artificial Neural Networks.

[56] Li W., Huang X., Zhu Z., Tang Y., Li X., Zhou J., Lu J. (2022). Ordinalclip: Learning rank prompts for language-guided ordinal regression. Adv. Neural Inf. Process. Syst..

[57] Cheng Y., Ying H., Hu R., Wang J., Zheng W., Zhang X., Chen D., Wu J. (2023). Robust image ordinal regression with controllable image generation. arXiv.

[58] Wang R., Li P., Huang H., Cao C., He R., He Z. (2023). Learning-to-rank meets language: Boosting language-driven ordering alignment for ordinal classification. Adv. Neural Inf. Process. Syst..

[59] Jiang S., Ji Q., Shi H., Chen C.Y., Xu Y. (2025). Spatial correlation guided cross scale feature fusion for age and gender estimation. Sci. Rep..

